# Supplementary material for: Evidence on the links between water insecurity, inadequate sanitation and mental health: A systematic review and meta-analysis
Source: PLoS One. 2023 May 25;18(5):e0286146. doi: 10.1371/journal.pone.0286146 (PMC10212143; doi:10.1371/journal.pone.0286146)
Supplement: S1 Appendix — (DOCX) [file pone.0286146.s002.docx]

**EMBASE**

Embase <1974 to 2023 March 21>

1 exp mental health/ 226798

2 exp water supply/ or exp water insecurity/ 45320

3 exp sanitation/ 540514

4 ("mental health" or "mental disorders" or "common mental disorders" or depression or anxiety or "mental distress" or "well-being" or wellbeing).tw. 1083618

5 ("water *insecurity" or "water scarcity" or "access to water" or "water access" or "water availability" or "water supply" or "water quan*" or "water distribution" or sanitation or "open defecation" or "toilet facility*" or latrine or "poor sanitation").tw. 38239

6 1 or 4 1156232

7 2 or 3 or 5 556470

8 6 and 7 4854

**PsycINFO**

APA PsycInfo <1806 to March Week 2 2023>

1 exp Mental Health/ 86419

2 exp Water Deprivation/ 937

3 exp Hygiene/ 1920

4 ("mental health" or "mental disorders" or "common mental disorders" or depression or anxiety or "mental distress" or "well-being" or wellbeing).tw. 740714

5 ("water *insecurity" or "water scarcity" or "access to water" or "water access" or "water availability" or "water supply" or "water quan*" or "water distribution" or sanitation or "open defecation" or "toilet facility*" or latrine or "poor sanitation").tw. 1889

6 1 or 4 746595

7 2 or 3 or 5 4456

8 6 and 7 567

[**https://access.ovid.com/custom/redirector/wayfless.html?idp=https://kclidpdev.kcl.ac.uk/idp/shibboleth&url=http://ovidsp.ovid.com/ovidweb.cgi?T=JS&NEWS=N&PAGE=main&SHAREDSEARCHID=pGxkPg3UJwKjQmHFwGteBSDA51qoXXDjzbXr36Mc2ReJSVlCkyz7Hf20hh4wmnXj**](https://access.ovid.com/custom/redirector/wayfless.html?idp=https://kclidpdev.kcl.ac.uk/idp/shibboleth&url=http://ovidsp.ovid.com/ovidweb.cgi?T=JS&NEWS=N&PAGE=main&SHAREDSEARCHID=pGxkPg3UJwKjQmHFwGteBSDA51qoXXDjzbXr36Mc2ReJSVlCkyz7Hf20hh4wmnXj)

**PUBMED**

Search: **(("Mental Health"[Mesh]) OR ("mental health" OR "mental disorders" OR "common mental disorders" OR depression OR anxiety OR "mental distress" OR "well-being" OR wellbeing)) AND ((("Water Insecurity"[Mesh]) OR ("water *insecurity" OR "water scarcity" OR "access to water" OR "water access" OR "water availability" OR "water supply" OR "water quan*" OR "water distribution" OR sanitation OR "open defecation" OR "toilet facility*" OR latrine OR "poor sanitation")) OR ("Sanitation"[Mesh]))**

("Mental Health"[MeSH Terms] OR ("Mental Health"[All Fields] OR "mental disorders"[All Fields] OR "common mental disorders"[All Fields] OR ("depressed"[All Fields] OR "depression"[MeSH Terms] OR "depression"[All Fields] OR "depressions"[All Fields] OR "depression s"[All Fields] OR "depressive disorder"[MeSH Terms] OR ("depressive"[All Fields] AND "disorder"[All Fields]) OR "depressive disorder"[All Fields] OR "depressivity"[All Fields] OR "depressive"[All Fields] OR "depressively"[All Fields] OR "depressiveness"[All Fields] OR "depressives"[All Fields]) OR ("anxiety"[MeSH Terms] OR "anxiety"[All Fields] OR "anxieties"[All Fields] OR "anxiety s"[All Fields]) OR "mental distress"[All Fields] OR "well-being"[All Fields] OR "wellbeing"[All Fields])) AND ("Water Insecurity"[MeSH Terms] OR ("Water Insecurity"[All Fields] OR "water scarcity"[All Fields] OR "access to water"[All Fields] OR "water access"[All Fields] OR "water availability"[All Fields] OR "water supply"[All Fields] OR "water quan*"[All Fields] OR "water distribution"[All Fields] OR ("Sanitation"[MeSH Terms] OR "Sanitation"[All Fields] OR "sanitations"[All Fields] OR "sanitization"[All Fields] OR "sanitize"[All Fields] OR "sanitized"[All Fields] OR "sanitizer"[All Fields] OR "sanitizers"[All Fields] OR "sanitizing"[All Fields]) OR "open defecation"[All Fields] OR "toilet facility*"[All Fields] OR ("toilet facilities"[MeSH Terms] OR ("toilet"[All Fields] AND "facilities"[All Fields]) OR "toilet facilities"[All Fields] OR "latrine"[All Fields] OR "latrines"[All Fields]) OR "poor sanitation"[All Fields]) OR "Sanitation"[MeSH Terms])
