# Supplementary figures and images for: Evidence on the links between water insecurity, inadequate sanitation and mental health: A systematic review and meta-analysis
Source: PLoS One. 2023 May 25;18(5):e0286146. doi: 10.1371/journal.pone.0286146 (PMC10212143; doi:10.1371/journal.pone.0286146)

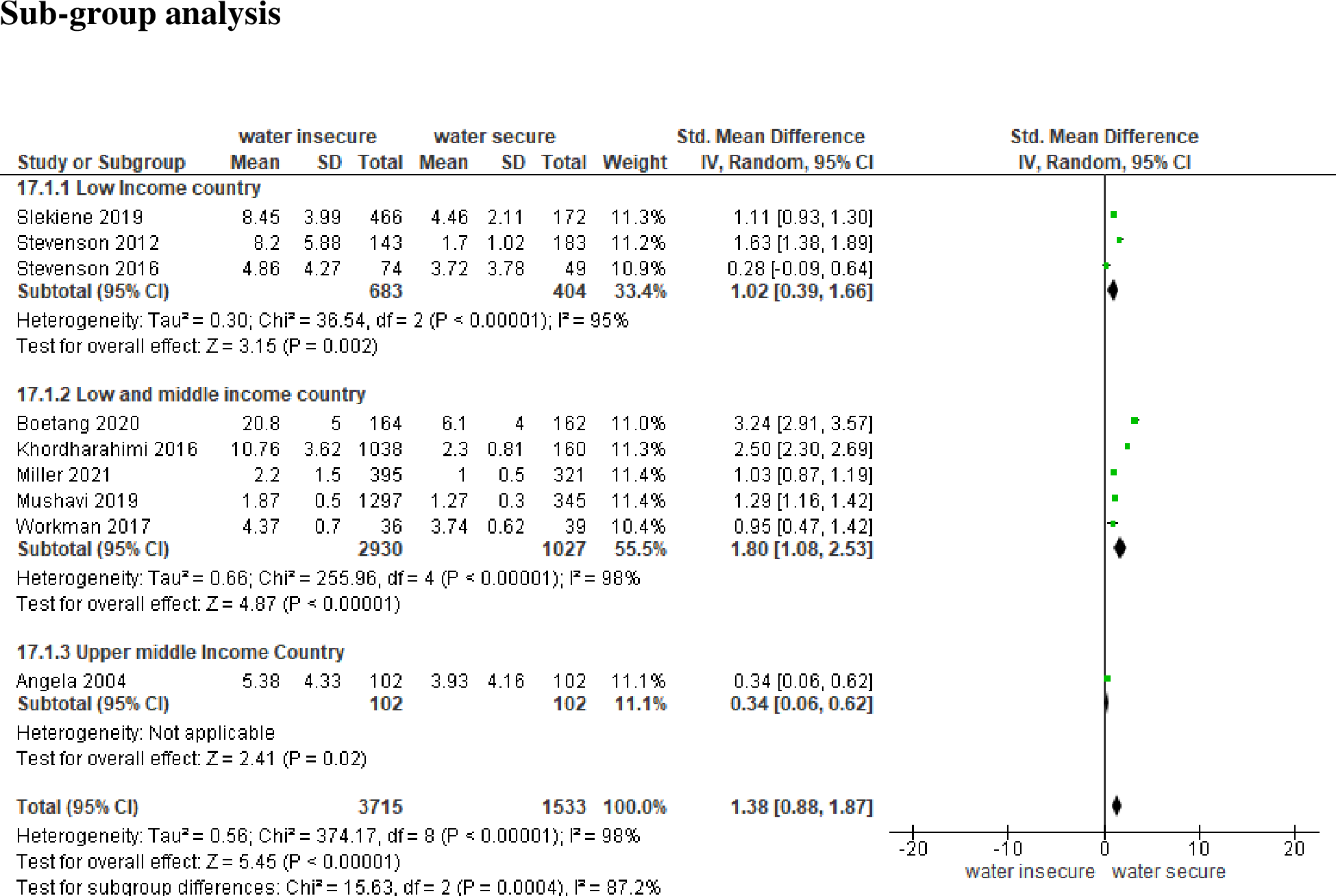

Supplement: S1 Fig — (TIF) [file pone.0286146.s005.tif]

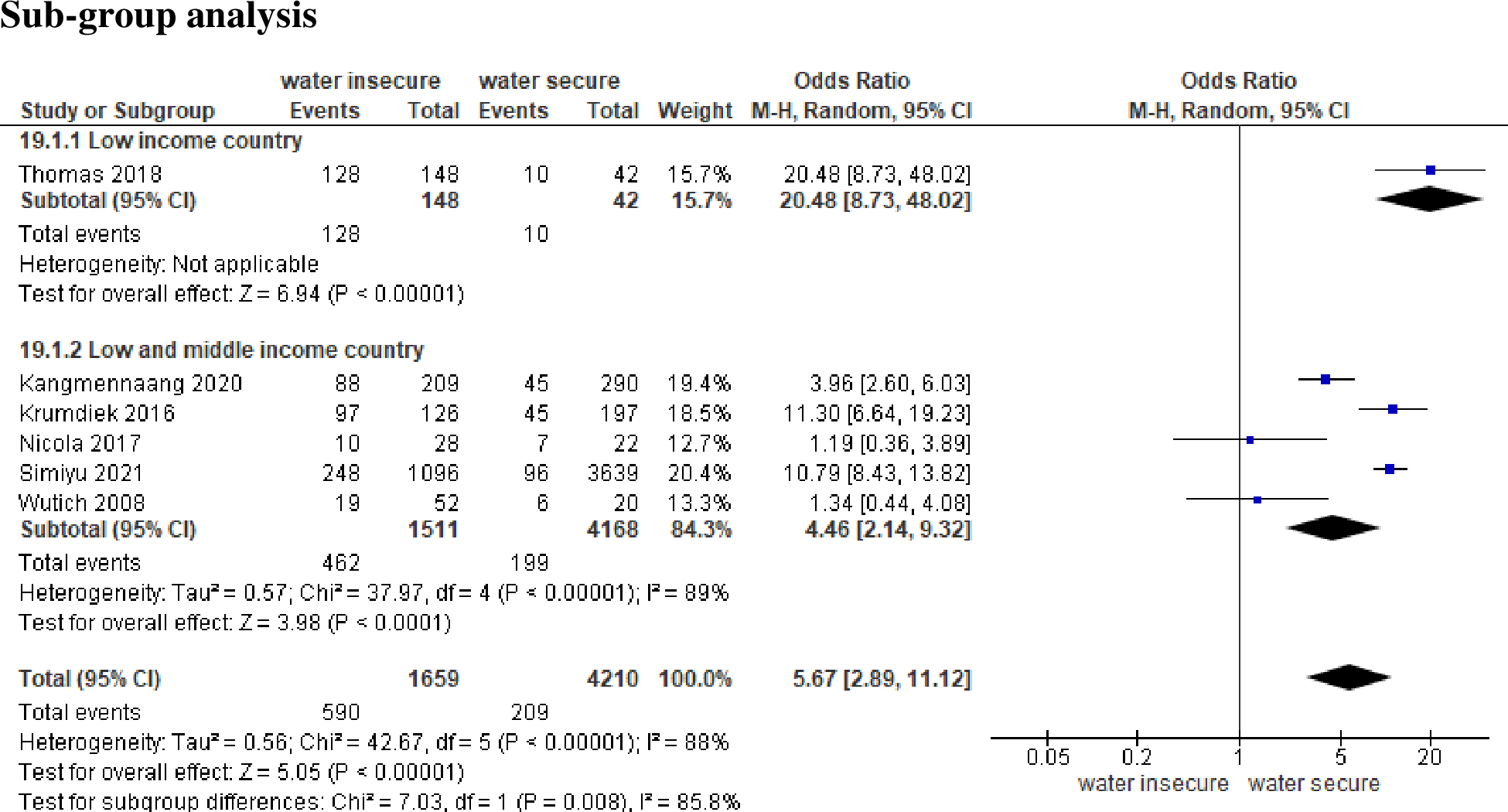

Supplement: S2 Fig — (TIF) [file pone.0286146.s006.tif]

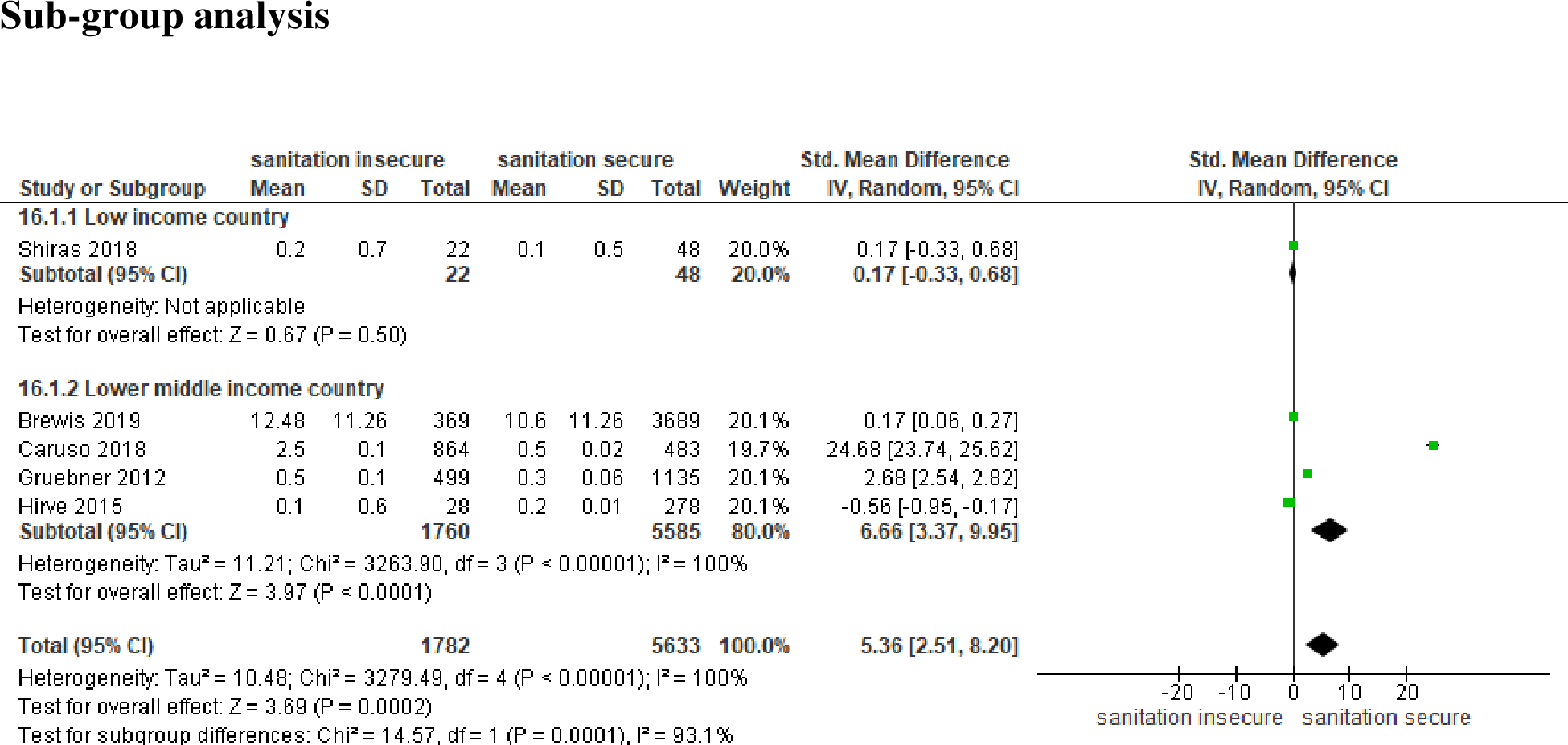

Supplement: S3 Fig — (TIF) [file pone.0286146.s007.tif]

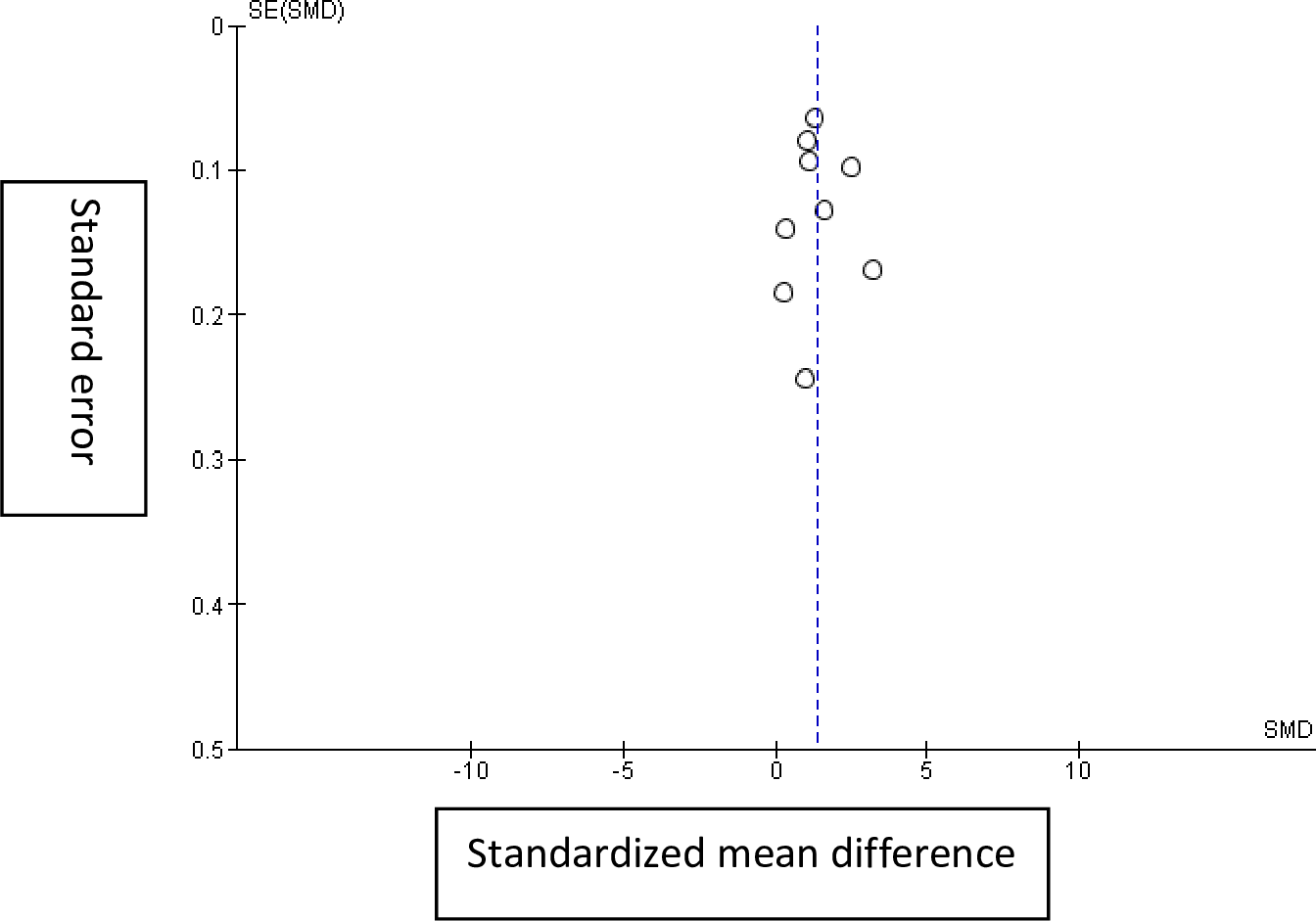

Supplement: S4 Fig — (TIF) [file pone.0286146.s008.tif]

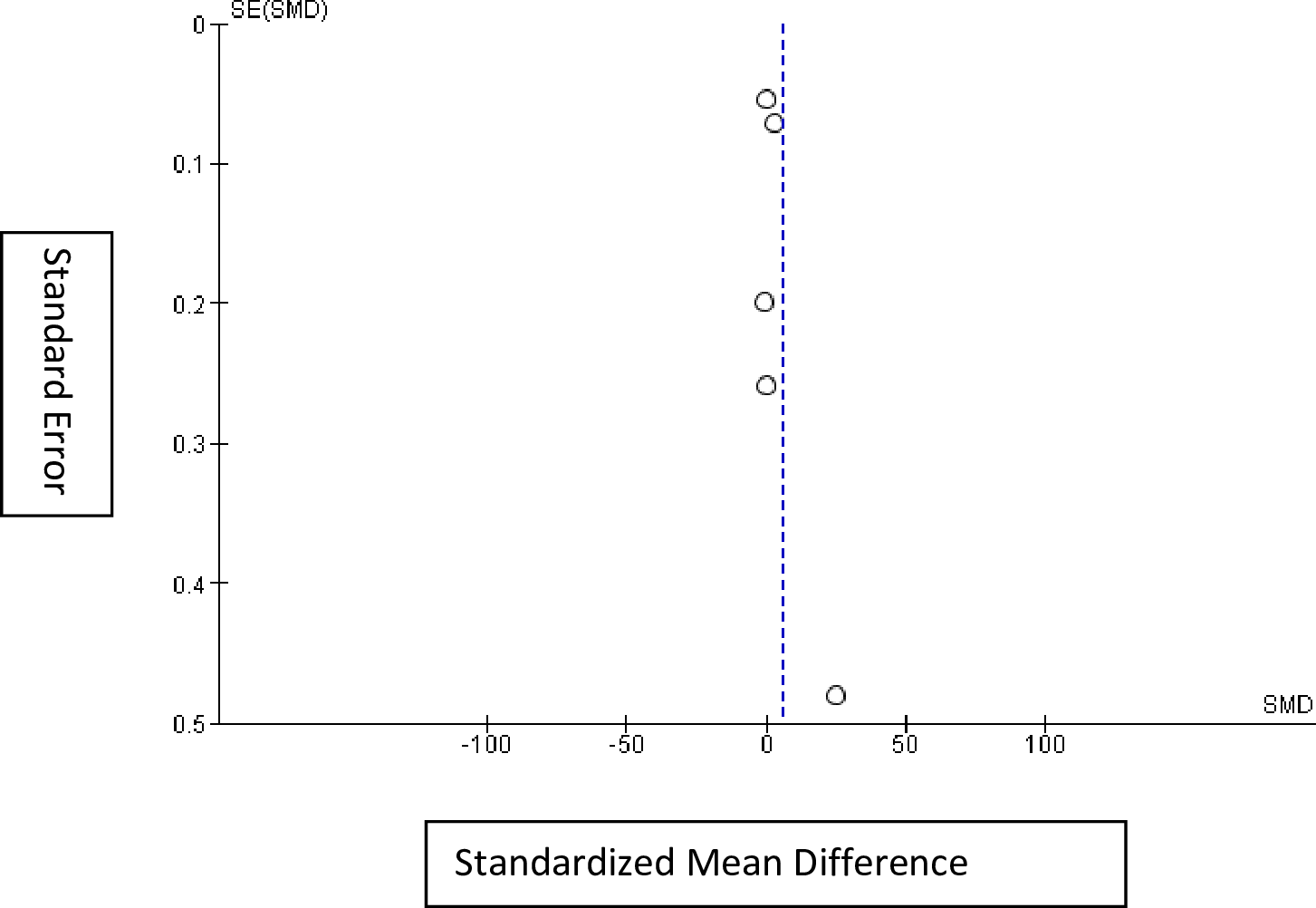

Supplement: S5 Fig — (TIF) [file pone.0286146.s009.tif]

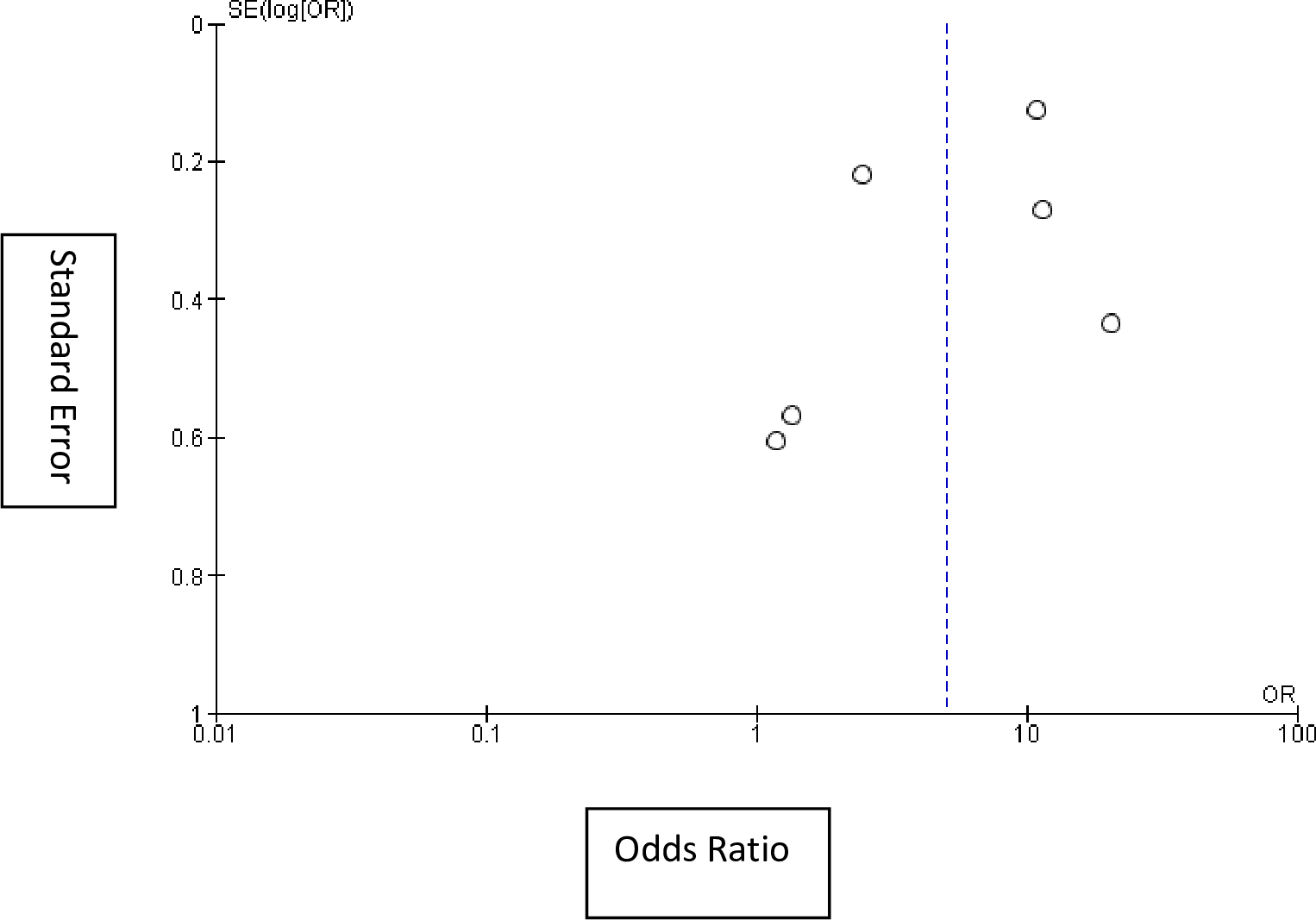

Supplement: S6 Fig — (TIF) [file pone.0286146.s010.tif]

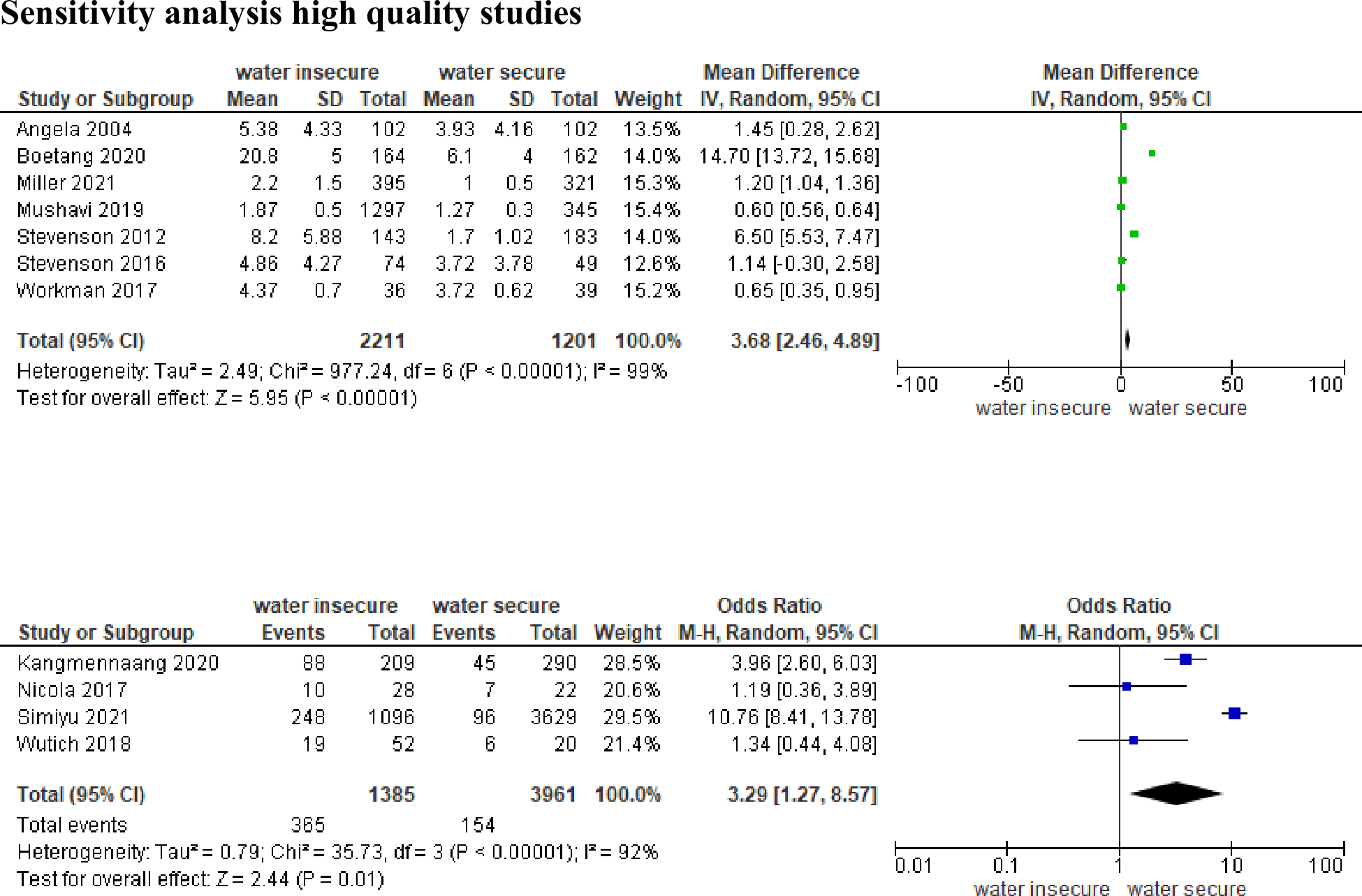

Supplement: S7 Fig — (TIF) [file pone.0286146.s011.tif]

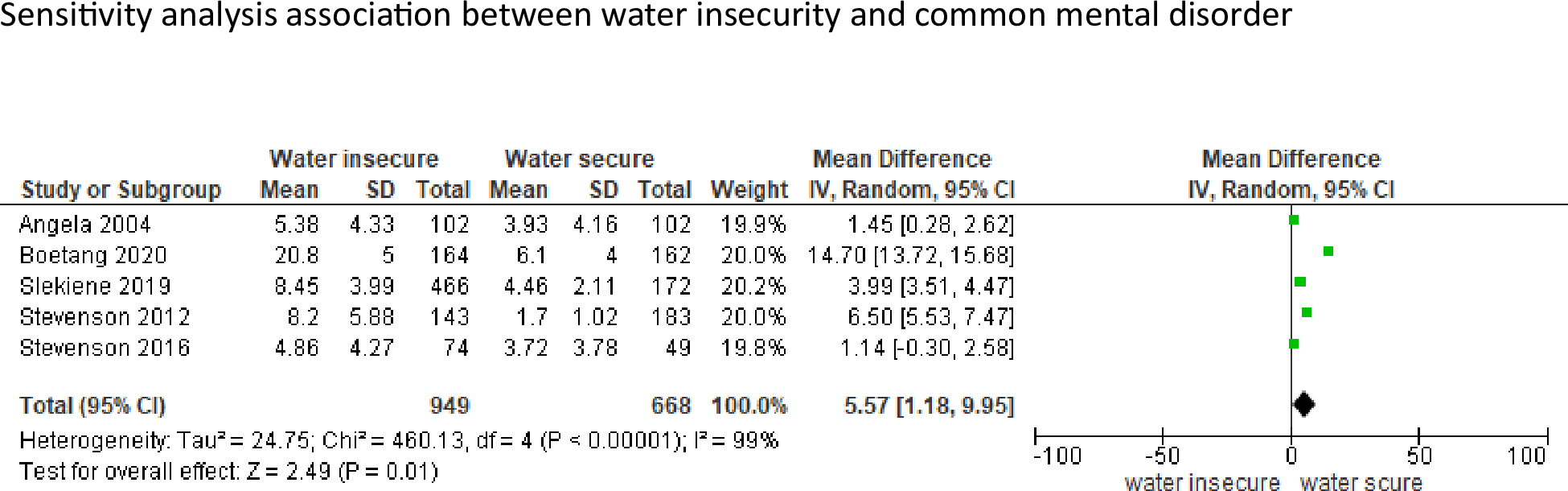

Supplement: S8 Fig — (TIF) [file pone.0286146.s012.tif]
